# Eye manifestations in Huntington’s disease: an update on the potential of ocular biomarkers

**DOI:** 10.1007/s00415-025-13600-4

**Published:** 2026-01-08

**Authors:** William A. Woods, Roger A. Barker

**Affiliations:** John Van Geest Centre for Brain Repair, Department of Clinical Neurosciences, University of Cambridge, Forvie Site, Cambridge, CB2 0PY UK

**Keywords:** Huntington’s disease, Ocular biomarkers, Retinal imaging, Optical coherence tomography, Neurodegeneration, Eye-movement abnormalities

## Abstract

Huntington’s disease (HD) remains a devastating neurodegenerative disorder caused by CAG repeat expansion in the HTT gene. Biomarkers are urgently needed to facilitate more accurate evaluation of disease onset, progression, and response to interventions. Characteristic clinical features of the disease are secondary to neuronal dysfunction, and the eye provides a potential window to characterize these changes. In this review, we systematically evaluate clinical studies examining ocular abnormalities in HD, including oculomotor function and retinal anatomy assessed by optical coherence tomography. Findings indicate that while ocular abnormalities can be identified in HD, their clinical utility remains unclear. Further evaluation in large cohorts of gene-positive individuals followed longitudinally is required.

## Introduction

Huntington’s disease (HD) is a genetic, progressive neurodegenerative disorder affecting approximately 1 in 7,300 individuals in Western populations, with many more being at risk of inheriting the gene [1–4]. The disease typically becomes manifested in peak adult life and includes motor, cognitive, and neuro-psychiatry disturbances, progressing over about two decades to death [5, 6]. Although early symptomatic treatments exist, no disease altering therapies are currently available.

Accurate biomarkers are required to diagnose and track disease progression as well as assess emerging therapies. Current clinical rating scales lack sensitivity in pre-manifest (pre-HD) or prodromal stages of HD limiting individualized prognosis to CAG repeat length and age [7, 8]. Potential biomarkers in HD, such as serial MRI and cerebrospinal fluid assays, face logistical and clinical challenges, while blood-based markers remain un-validated [9–12].

The ophthalmic system is affected early in HD and could be a potential non-invasive biomarker [13–15]. The retina is an accessible extension of the brain sharing embryological origin, structure and cellular composition which may reflect neurodegenerative changes in HD. Spectrum-domain optical coherence tomography (OCT) enables reproducible measurements of the retina including the retinal nerve fiber layer (RNFL) and ganglion cell-inner plexiform layer (GC-IPL) corresponding to ganglion cell axons and dendrites [16]. OCT for biomarker development is being evaluated across other neurodegenerative conditions, such as Parkinson’s, Alzheimer’s and multiple sclerosis [17–22].

Oculomotor assessment represents another promising biomarker domain. Abnormalities, such as saccadic and smooth pursuit eye movements, may precede other motor signs of HD and progress with disease severity [23]. These parameters can be measured objectively and are less subjective and effort-dependent than limb motor tasks, though longitudinal data is scarce and interindividual variability remains a limitation. Additional studies have described altered visual acuity, color discrimination, and electro-retino-graphic responses, suggesting broader ocular involvement in this condition.

This review systematically evaluates ocular biomarkers in HD, focusing on OCT and oculomotor findings, with reference to other visual and electrophysiological changes, to determine their potential value for diagnosis, prognostication, and clinical trial application.

## Methods

A systematic search was conducted utilizing the preferred reporting items for systematic review and meta-analysis (PRISMA) 2020 checklist and guidance [24, 25].

### Data collection and extraction

A systematic search was conducted in three electronic databases (Medline, Scopus, Cochrane library) from their inception until 29 October 2024. Search terms were focused on “Huntington’s disease” including related terms, such as “Pre-manifest” and the “Ocular” system, with related terms of “oculomotor”, “vision”, “eye”, “retina”, “OCT”. Searches included synonyms and related words. Reference sections were also searched for additional related studies not identified in the electronic searches.

Included studies recruited genetically confirmed HTT carriers with a CAG repeat above 38 at any stage of disease including pre-manifest and were compared to healthy controls. Studies characterizing the ocular system in HD were selected for inclusion. Only human studies published in English language were included. Studies reporting cases without genetically confirmed HD were excluded. Studies focusing on visuospatial memory or functioning were not included. Review articles were excluded but relevant meta-analysis included.

Duplicate articles were manually removed before abstracts, titles and then full texts were scrutinized prior to inclusion. A single investigator extracted data based on the type of study being examined. Studies were grouped broadly into categories of the primary focus of the research to allow for comparison and observation of replicated or inconsistent findings throughout the literature. A summary narrative was performed without meta-analysis.

### Quality assessment

The Mixed Method Appraisal Tool (MMAT) was utilized to characterize the quality of included studies presenting empirical data [26]. The MMAT was designed to allow critical appraisal for systematic reviews of mixed method articles, such as qualitative research, randomized control trials, non-randomized studies, quantitative descriptive studies, and mixed methods studies.

## Results

### Study selection

**Fig. 1:**
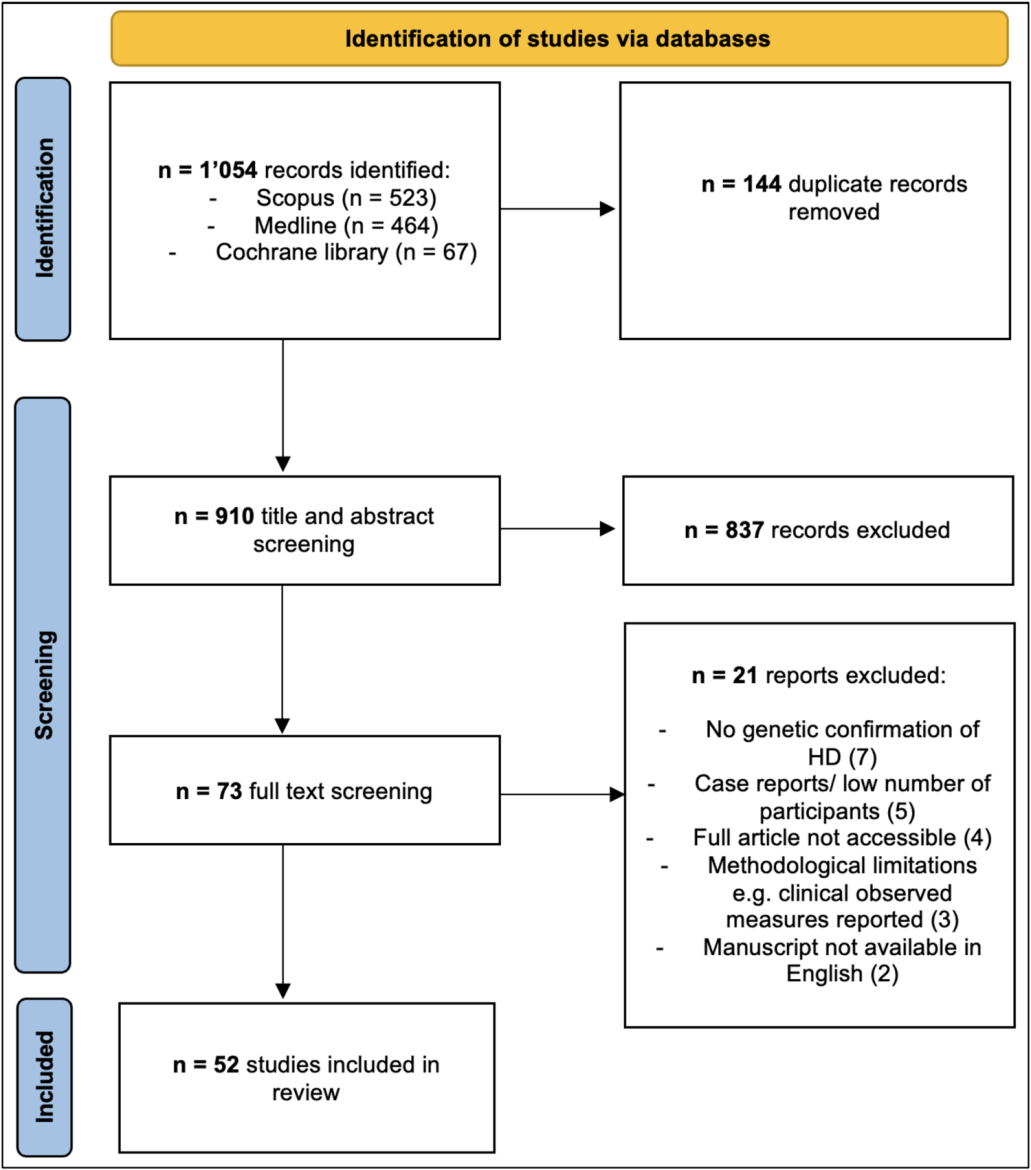
flow chart of study selection process

The study selection process is outlined in Fig. 1. A total of 1,054 articles were retrieved from electronic database searches (Scopus 521, Medline 464, Cochrane library 67). Duplicate articles were manually removed leaving 910. Title and abstracts screening excluded 837 articles with the most common reasons being: incorrect focus of study, non-HD population studied or incorrect article type such as reviews. The remaining 73 studies underwent full text screening and 19 excluded for reasons detailed in Fig. 1 leaving 52 unique studies for inclusion. One study, published in Russian, examining 44 HD participants with OCT was excluded as the same authors published a larger very similar study the following year which was deemed highly likely to include the same patient cohorts.

### Study characteristics and ocular themes in HD

Studies were grouped into themes based on the ocular parameter examined and methodological approach. OCT measurements were undertaken in 13 studies. Eye movements were reported in 19 studies where infrared devices were utilized for eye-tracking in all studies other than single investigations with magnetic scleral search coils and electrooculography (EOG). Single studies reported findings on visual acuity, color discrimination and retinal incremental thresholds and contrast sensitivity. Electrophysiological investigation was conducted in five studies; visual-evoked potentials in four studies and electroretinogram in a single.

### Optical coherence tomography

OCT is a gold-standard technique for the evaluation of retinal anatomy and is widely available [19]. The retinal nerve fiber layer (RNFL) and Ganglion Cell-Inner Plexiform Layer (GC-IPL) represent the axons and the cell bodies with dendrites of the retinal ganglion cells, respectively. Evaluation of the peripapillary RNFL (pRNFL), centered on the optic nerve head, and the ganglion cell complex (GCC)—comprising the macular RNFL and GC-IPL centered on the fovea—has been a particular focus in studies of neurodegeneration [27]. The choroidal layers of the retina, which represent distal branches of the internal carotid artery, have also been characterized. In contrast to spectrum domain-OCT (SD-OCT) which provides structural information, OCT angiography (OCTA) enables assessment of blood flow within the retinal microvasculature and choriocapillaries.

SD-OCT or OCTA was used in 13 HD studies as summarized in Table 1. Similar exclusion criteria including ophthalmic and systemic co-morbidities were deployed across studies with some additionally excluding patients based on ocular parameters, such as intra-ocular pressure and acuity. There was variability in the OCT model/manufacturer and analysis techniques (single eye vs both vs average). Most studies were undertaken on Western populations and all were cross-sectional case–control studies comparing HD to control participants at a single timepoint.

**Table 1:** OCT investigations in Huntington’s disease

Study	Participants (eyes included in analysis)	Mean age (y)	Gender (M %)	UHDRS-TMS	OCT parameters examined	Significant retinal changes reported in their HD cohort
Kersten et al. (2015)	20 manifest HD (20 eyes)^*^ 6 pre-manifest (6 eyes) 29 controls (28 eyes)	53.9 46.0 50.7	55 33 38	NR	• pRNFL thickness • Macular retinal volume and thickness	o Temporal pRNFL thinning (in manifest HD and a combined pre and manifest HD group analysis)
Andrade et al. (2016)	8 manifest HD (8 eyes) 8 controls (8 eyes)	49.1 49.8	38 38	31.5 ± 16.4	• pRNFL thickness • Peripapillary choroid thickness • Macula retinal thickness • Macula choroid thickness	o Average, central and inferior macula choroid thinning
Gatto et al. (2018)	14 manifest HD (27 eyes) 13 controls (26 eyes)	48.1 NR	36 NR	NR	• pRNFL thickness	o Temporal and superior pRNFL thinning o Average pRNFL thinning in a subgroup analysis of stage 3 HD (no overall thinning in HD)
Gulmez Sivem et al. (2019)	15 manifest HD (15 eyes) 15 controls (15 eyes)	NR NR	60 60	20.7 ± 2.0	• pRNFL thickness • Macular retinal thickness and volumes including single layers measurements	o Temporal pRNFL thinning (including superior and inferior temporal zones) o Macula retinal layer abnormalities: ▪ Layers thinned: RNFL, GCL, IPL, INL, OPL ▪ Layers thickened: ONL, outer retinal layer ▪ Volume reduced: IPL, RPE, outer macula
Di Maio et al. (2021)	16 manifest HD (32 eyes) 13 controls (26 eyes)	57.3 55.0	81 62	29.7 ± 12.9	• pRNFL thickness • Macula GCC thickness • Central choroid thickness • Vessel density of superficial and deep plexus capillary and choriocapillaris	o Central macula choroid thinning
Svetozarskiy et al. (2020)	31 manifest HD (31 eyes) 29 pre-HD (29 eyes) Controls 31 (31 eyes)	42.6 30.6 37.3	NR NR NR	36.3 ± 29.7	• pRNFL thickness • Macula GCC thickness • Central choroid thickness	o Average and temporal pRNFL thinning (pre and manifest HD) ▪ Nasal and inferior pRNFL additionally thinned in manifest HD o GCC thinning (pre and manifest HD) o Central macula choroid thinning (pre and manifest HD)
Mazur-Michalek et al. (2022)	13 pre-HD (26 eyes) 14 controls (28 eyes)	43.1 39.9	NR NR	Below 5 for all pre-HD	• pRNFL thickness • Macular GCC thickness • Optic nerve cup and disk area and volume	o Average, superior, inferior and temporal (right eye only) pRNFL thinning
Amini et al. (2022)	25 manifest HD (46 eyes) 25 controls (50 eyes)	49.8 44.3	56 40	29.3 ± 17.8	• pRNFL thickness • Macular retinal thickness • Superficial and deep capillary plexus vessel density	o Inferior pRNFL thinning o Macula retinal thinning in the inner superior and outer superior and inferior zones
Schmid et al. (2022)	24 pre-HD far from onset ^**^ 38 controls ^**^	39.7 35.8	42 37	3.3 ± 3.9	• Macular thickness including RNFL, GCL and GCIPL	o Inner macula RNFL thickness increased in pre-HD
Dusek et al. (2023)	41 manifest HD (82 eyes) 41 controls (82 eyes)	50.6 48.2	51 46	30.0 ± 12.3	• pRNFL thickness • Macular retinal volume	No statistically significant findings passed false discovery testing. Initially significant with small effect sizes: o Mean RNFL thickness and macula volume
Murueta-Goyena et al. (2023)	20 manifest HD (38 eyes) 16 pre-HD (32 eyes) 20 manifest controls (40 eyes) 16 pre-HD controls (eyes 32)	53.4 46.0 53.2 46.0	45 25 45 25	29.0 ± 2.0 1.75 ± 2.0	• pRNFL thickness • Macular retinal complex layer thicknesses	o Temporal pRNFL thinning in manifest HD o Macular and perifoveal ELM-BM complex thinning (pre and manifest HD)
Joseph et al. (2024)	23 HD (19 manifest and 4 prodromal, 44 eyes) 38 controls (76 eyes)	54.9 56.9	43 50	NR	• pRNFL thickness • Macula thickness including GCIPL • Choroid vascularity index • FAZ area • Superficial capillary plexus vessel and perfusion density • Radial peripapillary capillary plexus perfusion density and flux index	o GCIPL thinning o Decreased FAZ area
Shah et al. (2024)	12 manifest HD (16 eyes) 16 controls (31 eyes)	52.5 75.4	NR NR	NR	• Superficial and deep capillary plexus at the fovea • FAZ area	o Reduced vessel density in the superficial capillary plexus at the fovea o Increased vessel density in the deep capillary plexus at the fovea

*HD*, Huntington’s disease; *pre-HD*, pre-manifest HD; *NR*, not reported; *pRNFL*,; peripapillary retinal nerve fiber layer; *GCL*, Ganglion Cell Layer; *IPL*, Inner Plexiform Layer; *INL*, Inner Nuclear Layer; *OPL*, Outer Plexiform Layer; *ONL*, Outer Nuclear Layer; *RPE*, Retinal Pigmented Epithelial; *GCC*, Ganglion Cell Complex; *GCIPL*, Ganglion Cell Inner Plexiform Layer; *FAZ*, Foveal Avascular Zone. Average pRNFL refers to the mean of the total area across all quadrants

Summary of HD OCT studies. The frequency of eyes indicates distinct anatomic eyes analyzed by studies

^*^up to four participants excluded from either RNFL and/or macula due to low scan quality

^**^unclear if left and/or right eyes were analyzed

Temporal pRNFL thinning in manifest HD was the most consistent significant finding but reported in only 5 out of the 10 studies that looked at this [28–32]. In a study of 15 manifest HD patients, temporal pRNFL thinning negatively correlated with disease burden score, CAG count, disease duration, UHDRS total motor score (TMS) and independence scale [30]. This was not consistently replicated although two further studies also reported temporal thinning correlations with TMS and disease duration respectively [28, 31]. Five studies reported no significant temporal pRNFL thinning in HD including the largest study of 41 patients [33–37]. Further pRNFL quadrant thinning in the superior, inferior and nasal sectors were reported each in two studies [29, 31, 35]. The average pRNFL (mean across all quadrants) in manifest HD was reported to be thinned in a single study and found to correlate with disease burden and CAG length [31]. A further study reported average pRNFL thinning in a subgroup of stage 3 HD patients although overall there was no thinning in HD patients compared to controls [29]. Similar trends of average pRNFL thinning were described but did not reach statistical significance or pass false discovery testing in two further studies [32, 36]. Four studies reported no significant pRNFL abnormalities in manifest HD [33, 34, 36, 37].

Pre-HD pRNFL was examined in four studies with significant abnormalities reported in two [28, 31, 32, 38]. A single study reported increased eye asymmetry in pre-HD with thinning of the average, superior, inferior, and temporal quadrants of which the latter was significant only in the right eye [38]. A further study with a relatively poorly age matched control cohort reported average and temporal retinal thinning [31].

The retinal macula was examined in eleven studies [28, 30–39]. There was limited evidence for changes in the overall macular thickness and volume from four manifest and two pre-HD cohorts [28, 30, 34, 36, 39]. A single peer reviewed study and a conference abstract reported overall superior macula thinning and macula volume reduction respectively in manifest HD [35, 40]. Segmentation of the macula into specific cell layers or complexes differed between studies and there was little consistency in findings across five studies showing abnormalities in HD (Table 1). A significant proportion of studies reported no macula layer changes in pre or manifest HD [28, 33, 34, 36, 38, 39].

Regarding studies reporting on GCC thicknesses, a single study reported thinning in pre and manifest HD but this was not corroborated by other studies [31, 33, 38]. When the GC-IPL complex thickness was reported, there was no significant thinning in manifest or pre-HD [32, 37, 39]. In one of these studies, GC-IPL thinning became significant when the manifest group was combined with a prodromal group of patients [37]. The ganglion cell layer was segmented in single studies of pre and manifest disease with the latter being reported as showing thinning [30, 39]. A variety of further abnormalities were reported in HD in individual studies involving the external limiting membrane—Bruch’s membrane complex and individual layers of the macula (Table 1) [30, 32, 39].

The central macula choroid has been consistently reported to be thinned in HD [31, 33, 34]. However, one of these studies may have been methodologically inaccurate with manual segmentation of the choroid using SD-OCT without enhanced depth or a swept source [31]. A single study examined non-central macula choroid measurements reporting thinning in the average and inferior sectors in 8 HD participants [34]. One study calculated the choroid vascular index (CVI) representing the proportion of the luminal area in the macula choroid and found no difference in HD [37]. OCTA was utilized in four manifest HD studies to characterize retinal vascularity [33, 35, 37, 41]. All but one of these reported no differences in capillary plexus vessel densities [33, 35, 37]. The remaining study of 12 manifest participants reported a lower mean superficial foveal capillary density and greater deep foveal capillary density in HD [41]. The area of the foveal avascular zone was reported in two studies and found to be reduced in one with a non-significant trend in the other [37, 41]. No studies have utilized OCTA in pre-manifest groups.

### Ocular movements

Ocular movements stabilize images on the retina (fixation, smooth pursuit, vestibulo-ocular and optokinetic reflexes) or direct vision toward new stimuli (saccades) [42]. The former are largely automatic, whereas the latter require voluntary control or involvement of higher cognitive processing [43–45]. Clinical examination alone lacks sensitivity to detect subtle abnormalities in HD, such as those occurring in the pre-HD phase [23, 46–50]. Quantitative techniques including infrared tracking, EOG and the scleral search coils have improved detection offering the potential for biomarker development [51–61].

This section summarizes oculomotor dysfunction in HD including abnormalities in saccades, visual scanning, optokinetic nystagmus and distractibility (Table 2) [51, 53–56, 58, 59, 62–68]. Studies prior to genetic confirmation or lacking quantitative assessments are not discussed in detail. Nevertheless, earlier observations of prolonged saccade latency, decreased velocity, initiation with head movements or blinking, distractibility, and smooth pursuit abnormalities have all helped define this area for biomarker development [14, 15, 69–76].

**Table 2:** Oculomotor characterization in Huntington’s disease by eye-tracking technique

Study	Participants	Mean age (y)	Gender (M %)	UHDRS-TMS	Summary of movement tasks investigated	Significant oculomotor abnormalities in HD
Magnetic scleral search coil technique						
Winograd-Gurvich (2003)	11 manifest HD 11 controls	51.3 51.3	64 64	31.1	PS VGS: - Predictive task - Self-paced speed switching task	Manifest HD: • Prolonged latency for larger amplitude saccades in predictive tasks • Inaccuracy with hypo-metric saccades requiring more subsequent corrective saccades during PS and self-paced tasks • Increased inter-saccadic intervals and variability in self-paced tasks
Electrooculogram + infrared eye-tracking						
Peltsch (2008)	9 manifest HD 9 controls	51.9 52.4	67 67	27.3	EOG tracking tasks: - PS - AS Infrared tracking tasks: - MGS	Manifest HD: • Prolonged PS and AS latency and increased variability • Greater errors in anti-saccades • Higher rate of early timing errors in the MGS delayed sequence task • Inability to perform MGS delayed sequence task successfully and saccades were less accurate • Correlations between UHDRS-TMS were most notable for anti-saccade error rates
Infrared eye-tracking						
Blekher (2004)	8 manifest HD 9 pre-HD 19 controls	50.4 49.7 50.4	63 44 36	NR NR	PS AS MGS Pursuit OKN Visual scanning	Manifest HD: • Increased latency and reduced velocity during PS, AS, and MGS tasks • Increased error rates in AS and MGS • Reduced OKN gain • Less exploratory saccades in the visual scanning task Pre-HD: • Increase error rates during the MGS compared to controls
Ali (2006)	24 gene-positive HD (pre and manifest) 20 controls	59.3 47.7	38 55	20.5 NR	PS	• Increased median latency, duration and variability compared to controls • Increased frequency of early saccades
Golding (2006)	12 manifest HD 12 pre-HD 12 manifest HD controls 12 pre-HD controls	47 35 49 34	17 33 17 66	13.5 < 5	PS VGS: - Central cueing	Manifest HD: • Prolonged latency (PS, VGS) and increased variability compared to pre-HD • Reduced velocity (PS, VGS) which correlated inversely with disease severity Pre-HD: • Prolonged latency in VGS compared to controls
Blekher (2006)	30 manifest HD 16 prodromal HD 27 pre-HD 142 controls	50 44 45 47	40 44 41 22	26 ± 10 15 ± 6 5 ± 4 5 ± 4	AS VGS: - Predictive task - MGS Fixation with distractors	Manifest HD: • Increased errors in AS and MGS (progressive trend from pre < prodromal < manifest) • Prolonged latency in predictive, AS, MGS with increased variability in AS and MGS • Reduced fixation over 60 s Prodromal HD: • Increased error rates in AS and MGS • Prolonged latency in predictive, AS, MGS Pre-HD: • Increased errors in AS and MGS • Prolonged latency in MGS
Antoniades (2007)	12 manifest HD 18 Pre-HD 10 manifest HD controls 8 pre-HD controls	61.3 45.0 62.4 45.6	NR NR NR NR	20 2.4	PS	Pre-HD: • Increased incidence of early saccades (abnormally short latencies) compared to controls
Hicks (2008)	12 manifest HD^*^ 17 pre-HD^*^ 12 controls^*^	NR NR NR	NR NR NR	NR NR	PS VGS - First-order conditional task - Second-order condition task	LATER model plot comparisons (no formal statistical analysis to assess between group differences): - Error rates increased with task complexity which better differentiated disease groups - Early saccade incidence differences emerged between disease groups with increased task complexity
Robert (2009)	12 manifest HD^*^ 17 pre-HD^*^ 12 controls^*^	NR NR NR	NR NR NR	NR NR	PS VGS - First-order conditional task - Second-order conditional task	The second-order conditional task was able to differentiate disease groups based on saccadic latency distributions: • Manifest HD; prolonged latencies, high variability, increased difficulty handling incongruent trials • Pre-HD; abnormalities were less severe than the manifest group but significantly differed from controls Low rule following accuracy increased latency and variability
Tabrizi ^*^ (2009)	123 manifest HD 120 pre-HD 123 controls	NR NR NR	NR NR NR	NR NR	VGS - Second-order conditional task	Anti-saccade error rates increased proportionally across disease group stages: • Pre-HD over 10 years from onset (similar error rate to controls), Pre-HD within 10 years of estimate disease onset, early HD, more advanced HD (most errors)
Blekher (2009)	19 manifest HD 21 pre-HD 23 controls	51.5 51 51.1	26 33 35	33.3 ± 14.4 11.4 ± 7.1 1.1 ± 1.3	Visually scanning (during the digit symbol test)	• Visual scanning maneuvers decreased stepwise from control to Pre-HD to HD (despite number of saccades remaining stable) • Number of scanning maneuvers correlated with the digit symbol test score (scanning task) across HD and control groups
Antoniades (2010)	18 manifest HD 18 pre-HD 12 controls	55.2 45.5 50.1	56 56 58	27.1 1.9	PS	Progressive stepwise changes in saccades over 3 years in both pre and manifest HD groups: • Latency increased over time o Saccade initiation was faster in pre-HD than in manifest HD • Early saccades frequency rates decreased over time Controls saccades remained stable
Henderson (2011)	12 manifest HD 12 controls	56.8 56.7	NR NR	18.4 ± 8.6	Smooth pursuit (with and without visual distractors)	Manifest HD: • No statistically significant findings between overall HD and control groups o Increased distraction in a stage 2 HD group
Grabska (2014)	10 juvenile HD 10 controls	27.2 30.5	69 60	32.7 ± 20.9	PS VGS - Self-paced speed switching task - First-order conditional task	Juvenile HD: • Prolonged latency and reduced velocity in PS o Latency correlated with disease severity and inversely with working memory • Reduced frequency and amplitude of self-paces switching saccades • Increased errors in first-order conditional tasks
Miranda (2016)	14 manifest HD 14 pre-HD 22 controls	44.9 35.8 36.2	29 43 32	26.5 1.92	PS AS MGS - 1 or 2 back PS - 1 or 2 back AS	Machine learning (single-vector machine) was utilized across the different oculomotor tasks to assess classification of disease groups - Classification performance for a given oculomotor features was influenced by disease stage and task type - Classification accuracy achieved: o Control vs pre-HD: 73% o Control vs manifest HD: 82% o Pre-HD vs manifest HD: 84%
Kordsachia (2017)	25 gene-positive HD (pre and early manifest) 25 controls	49.4 49.6	44 40	8.3 ± 9.2	Visual scanning (emotionally provocative images)	• Lower proportion of viewing time spent fixating • Shorter average fixation durations • Longer scanning paths
Vaca-Palomares (2019)	22 manifest HD 23 controls	49.6 49.9	41 43	17.0 ± 12.6	PS VGS - Predictive task	Manifest HD: • Predictive abnormalities; longer saccadic latencies at the start of the task and more repetitions before latency shortening appeared o HD with high TMS had less ability to anticipate • Greater saccade amplitude in predictive and reactive tasks compared to controls (overshooting)
Julio (2019)	15 pre-HD 22 controls	NR (median = 37) NR (median = 34)	47 32	< 5	PS AS MGS - 1 or 2 back PS - 1 or 2 back AS	Pre-HD: • Higher error rate for AS and MGS-AS tasks • Shorter latency time in MGS-AS tasks
Belardinelli (2021)	18 pre-HD^**^ 18 controls	35.6 37.3	50 50	5.8 ± 2.2 0.7 ± 1.2	Visual scanning (reading the mind in the eyes test)	Pre and prodromal HD group: • Reduced frequency of fixations • Shortened average fixation duration • Reduced time spent fixating on lower regions of images
Reyes-Lopez (2024)	22 manifest HD 23 controls	49.6 49.9	41 43	17.0 ± 12.6	Visual scanning (10 min video)	Manifest HD: • Reduced exploratory saccade rate following transitions to a new video • Higher blink rate throughout viewing • Increased pupil reaction to luminance changes after clip transitions

Summary of oculomotor studies in HD using eye-tracking methods. *HD*, Huntington’s disease; *EOG*, electrooculogram; *PS*, prosaccades (toward a peripheral target); *AS*, anti-saccades (saccade in the opposite direction to a peripheral target), *MGS*, memory guided saccades (maintain central fixation while a peripheral target is briefly flashed, retain its location during a delay, and then generate a saccade to the remembered position); *OKN*, optokinetic nystagmus; *VGS*, voluntary-guided saccades (non-reflexive saccades that are initiated according to an instruction; examples include MGS, predictive tasks where peripheral targets alternate predictively so testing anticipation, self-paced tasks where saccades are made between peripheral targets, first-order condition tasks, such as central cueing and second-order condition tasks, with multiple rules); *NR*, not reported. The Unified Huntington’s Disease Rating Scale—Total Motor Score (UHDRS-TMS) is represented for each study as the mean cohort score ± standard deviation when available

^*^Hicks et al. and Robert et al. likely studied the same cohorts

^**^pre-HD was defined as DCI < 4 and UHDRS-TMS ≤ 10

Prosaccades in manifest HD have prolonged but more variable latencies, as well as reduced velocities, compared with controls [53–56, 66, 77]. Abnormalities are typically more pronounced in anti-saccade tasks, where patients make more directional errors [54, 55, 66]. Increased cognitive load further impairs performances as seen in memory guided saccadic (MGS) tasks which show reduced accuracy to controls and exacerbated prolongation and variability in latencies [54, 55, 66]. Similar abnormalities in prosaccades have been reported in patients with Juvenile onset HD in a single study [78].

Saccade latencies vary widely within individuals and are not normally distributed. The distribution itself carries clinical relevance. The LATER model has demonstrated abnormal variability in HD, including an increased frequency of early saccades [49, 50, 56–58]. Some studies report more early saccades during simple prosaccade tasks in pre-HD [49, 50] although others have not replicated this finding using comparable paradigms [56]. Increased variability arises from a small proportion of saccades which occur early and are superimposed on generally slowed initiation. In longitudinal analysis, prosaccade latency increased and early saccade rates decreased over three years in both pre-HD and manifest HD, with no change in control [49].

In more complex oculomotor tasks, pre-HD exhibited increased anti-saccade error rates and prolonged latencies compared with controls [54, 55, 60]. MGS also showed reduced accuracy and increased latency though impairments are milder than in manifest and prodromal HD [54, 55]. A more complex MGS task combining two memory points and anti-saccade requirements also demonstrated increased error rates but paradoxically reduced latency in pre-HD suggesting impulsivity [60].

Cognitively demanding oculomotor tasks have been explored to determine whether oculomotor analysis during a complex task could allow for better HD stratification. Second-order conditional tasks (color cues directing pro- or anti-saccades toward shifting peripheral targets) distinguish pre-HD from manifest disease through error rates and prolonged latency [56, 57]. A larger study demonstrated progressive increases in error rates across disease stages—from far from onset pre-HD, to advanced HD [58].

Predictive saccade tasks, requiring anticipation of consistently alternating targets, show prolonged latency in manifest HD, particularly with larger target amplitudes [55, 63, 64]. Manifest HD also demonstrated delayed onset of anticipatory responses and reduced anticipatory accuracy with increasing UHDRS-TMS [63]. A single study in pre-HD found no significant latency changes, in contrast to prodromal and manifest groups [55].

Visual scanning paradigms have examined gaze behavior during functional tasks. In the digit symbol test, early work reported reduced saccade frequency in HD [54], whereas a larger follow-up study found a stepwise reduction in scanning maneuvers—but not saccade frequency—from pre-HD to manifest disease [52]. Reduced exploratory saccades have also been observed following transitions in video stimuli among manifest HD [62]. When viewing emotionally provocative images, HD gene carriers (pre-HD and early HD) show shortened fixation durations and longer scanning paths [59], and pre-HD participants demonstrate shorter and less frequent fixations when viewing emotional facial expressions [61]. Increased distractibility in manifest HD has been demonstrated in both trials of smooth pursuit and static fixation [55, 65].

### Other ocular manifestations in Huntington’s disease

Additional structural and functional changes have been described along the visual pathway in HD [79, 80]. Perceptual visual deficits in color and motion detection have been consistently reported in HD [28, 81, 82]. HD participants show impaired contrast sensitivity to moving targets and in visual acuity testing [81, 83]. Color discrimination deficits may occur before contrast sensitivity impairments in premanifest stages [84]. These abnormalities are clinically important as they may impact on real-life situations and increase vulnerability to accidents and injury. Such color vision impairment parallels findings in other neurodegenerative disorders, including Alzheimer’s and Parkinson’s disease [85, 86].

Visuospatial dysfunction in HD likely reflects impaired visuomotor integration [79, 87]. Psychomotor tests probing visual cognition demonstrate measurable deficits; for instance, the King–Devick rapid number-naming test shows a more than twofold increase in completion time in HD compared with controls [83].

Electrophysiological studies provide additional evidence of altered visual processing. A single electroretinogram study reported significantly increased amplitudes under both photopic and scotopic conditions, indicating possible cone pathway involvement [88]. Conversely, visual-evoked potential studies have described reduced cortical response amplitudes though none have been conducted in genetically confirmed cohorts [89–91]. The mechanism underlying increased retinal but decreased cortical responses remains unclear. An electroencephalography study in premanifest HD demonstrated reduced amplitude and delayed latency of preparatory potentials before anti-saccade execution, suggesting early disruption of cognitive control circuits despite preserved eye movement performance [92].

## Discussion

### Key findings

A variety of ocular biomarkers have been evaluated in HD, focusing primarily on retinal anatomy and oculomotor function. Reported retinal abnormalities were variable, while oculomotor studies have shown more consistent findings. However, no standardized assessment protocol has emerged to facilitate clinical translation.

### Optical coherence tomography findings

Retinal abnormalities may occur in HD, but current evidence from OCT studies remains limited and inconsistent. Temporal quadrant pRNFL thinning in HD was first reported in 2015 but has not been replicated by half of the subsequent studies. Macular findings have also been variable, and differences in segmentation techniques have restricted comparability.

Natural variability of retinal layer thickness within both disease and control groups limits clinical utility. Single timepoint OCT measurements are unlikely to distinguish between HD, premanifest HD, and controls patients. A recent meta-analysis suggested that changes may become significant at larger group levels with average and temporal pRNFL thinning in HD but not in pre-HD [93]. However, retinal abnormalities in premanifest HD have also been inconsistently reported and it remains unclear if thinning correlates with disease manifestation. For example, one study discussed in this review had a pre-HD group with more pronounced thinning of the temporal pRNFL than the manifest group. Conversely, retinal macular changes were not present in pre-HD far from onset as demonstrated in another study which instead reported caudate atrophy on MRI [39].

Participants in OCT studies typically represent early to moderate manifest HD, with an estimated mean disease duration of 7.9 years (weighted mean from five studies) and mean age of 50.3 years (weighted mean from nine studies). Due to chorea and cognitive impairment, OCT is unlikely to be feasible in advanced stages, and no data exists at this stage to determine whether abnormalities become more pronounced. There is, so far, little evidence to suggest pRNFL thinning correlates with clinical markers of advancing HD but this has not been properly evaluated. Post-mortem evidence of retinal involvement is also scarce, though one report of a 69-year-old patient with HD for 28 years found no retinal structural abnormalities [94].

### Limitations of OCT studies

Substantial methodological heterogeneity was evident across the studies. Segmentation methods ranged from automated with manual correction to unblinded manual segmentation, introducing potential bias. Some studies reported data from a single eye analysis despite collecting bilateral data. It is often unclear why. Right or left eyes were removed from the analysis after showing no statistically significant inter-eye differences using t tests; however, this reduced the sample size and lessened the statistical power of studies. Other studies have reported interocular asymmetry in HD and other neurodegenerative diseases [38, 95]. In a minority of studies, it was unclear how the statistical tests analysis had been performed. One study excluded a participant from OCT analysis after reporting statistics indicating there was no difference between HD and control group demographics.

Ocular vascular parameters also require further study after the initial findings of abnormalities in the foveal avascular zone and subfoveal choroidal thickness. However, choroidal measurements can fluctuate with intraocular pressure, diurnal variation, and potentially medication use, which clearly complicates the interpretation of any findings in this domain [96].

### Oculomotor function findings

Saccadic abnormalities in HD include increased variability, overall prolonged latency, reduced velocity, and decreased accuracy. These deficits are amplified by greater task complexity, larger saccades amplitudes, and higher cognitive load. Additional oculomotor impairments include reduced fixation stability, impaired visual scanning, and diminished anticipation ability. Although these abnormalities likely reflect frontostriatal dysfunction, the reasons for substantial inter-individual variability as seen with chorea and cognitive impairment, remains unclear [8, 97, 98].

Multiple oculomotor measures can distinguish between premanifest and manifest HD and healthy controls. Nevertheless, despite more than two decades of quantitative oculomotor research, no validated biomarker strategy has emerged. An ideal clinical tool would (1) reliably estimate time to disease onset, (2) support confirmation of disease stage, and (3) provide sensitive monitoring of disease progression. Current evidence does not support the first application, but limited data suggest potential utility for second and third.

Simple saccade task performance in pre-HD does not appear to correlate with established estimates of time to disease onset but warrants further investigation [49]. One cross-sectional study suggested that performance on more complex tasks may have predictive value, but this finding has not been confirmed longitudinally [57]. A limited number of studies indicate that oculomotor parameters may assist in staging, with machine learning approaches achieving accurate classification of pre-HD versus manifest HD (accuracy 83.5%) [50, 99]. For disease monitoring, the only longitudinal study to date reported progressive slowing of prosaccade initiation (increasing latency) and a reduction in the incidence of abnormally early saccades in both premanifest and manifest HD over three years [49]. Additional longitudinal investigations are required to evaluate and validate these biomarker strategies.

Visual scanning has become a more recent focus, with consistent abnormalities reported across multiple task paradigms. However, no biomarker-focused evaluation has yet been undertaken, and interpreting complex functional scanning tasks may pose methodological challenges.

### Limitations of oculomotor studies

The development of oculomotor biomarkers faces several challenges. Differences between disease stages in latency, fixation stability, and distractibility are typically small, necessitating specialized equipment that is not routinely available in outpatient clinics. Laboratory-based testing environments may also introduce confounding factors; devices such as the Saccadometer may offer advantages by enabling recordings in more natural settings [100]. Notably, most studies have focused almost exclusively on horizontal saccades, despite early evidence establishing that vertical saccades are disproportionally affected in HD [73–76].

Small but statistically significant intergroup differences may lack clinical relevance without longitudinal validation. Intra-individual variability in saccadic performance necessitates large numbers of repeated trials, yet individuals with HD may experience a disproportionate decline in attention and motivation across sessions. Standardized thresholds may therefore lack sensitivity for detecting stage-related differences. Performance may also fluctuate due to medication effects and mood, introducing further variability.

Uncertainty remains regarding whether cognitively demanding oculomotor paradigms offer advantages over established neuropsychological assessments of frontostriatal dysfunction, such as the Stroop test [101]. For example, in the Track-HD study, anti-saccade error rates increased across disease stages during a second-order conditional task, yet no corresponding timed oculomotor abnormalities (e.g., latency or peak velocity) were reported [58]. Functional oculomotor biomarkers, such as error rates, may prove more difficult to implement clinically than established cognitive tests and are similarly susceptible to external influences, limiting their objectivity.

Heterogeneity in study design and analytic approaches further complicates interpretation. Earlier studies reported prolonged latencies in HD, but the distribution of latencies only became clinically informative in later work involving substantially larger numbers of saccadic trials. Studies comparing all gene-positive individuals (premanifest and manifest) with controls can identify abnormalities but have limited value for biomarker development without disease stage-specific sub-analysis. Additionally, many studies excluded trials in which blinking occurred; however, increased blink frequency during saccade initiation is a recognized feature in advancing HD and its exclusion may remove clinically relevant data or could itself represent a potential biomarker.

### Clinical and research implications

Oculomotor assessments hold promise as objective, non-invasive measures of neurodegeneration in HD but require methodological refinement and longitudinal validation. OCT studies must determine whether retinal thinning really is there in HD, whether it progresses with disease severity and whether it predicts pheno-conversion. Likewise, oculomotor research should establish whether changes in saccadic latency, velocity, or accuracy correlate reliably with clinical or imaging markers of progression.

Longitudinal studies are essential to define the natural history of these ocular measures and to assess their responsiveness to disease-modifying interventions. To date, only a limited number of longitudinal oculomotor investigations have demonstrated meaningful temporal changes in saccadic parameters [49, 102].

For successful clinical translation, ocular biomarkers must either outperform or meaningfully complement existing measures. This includes demonstrating reproducibility across sites, resistance to confounding factors, and feasibility in routine clinical settings. Combining OCT and oculomotor metrics may enable a multimodal biomarker framework that reflects both structural and functional aspects of neurodegeneration. Machine learning approaches could facilitate such integration although their feasibility has not yet been assessed in combination. Additional modalities should also be explored; for example, rhodopsin sampling may represent a potential retinal marker of neurodegenerative and has yet to be evaluated in HD [103].

Despite extensive characterization of somatic CAG repeat expansion in vulnerable neuronal populations of the human brain, there has been no direct quantification of repeat length or mosaicism in human HD ocular tissues. As repeat instability in HD is highly specific to tissue and cell-type, defining somatic expansion in the eye is critical for interpreting OCT as limited local expansion would be unlikely to yield measurable structural pathology.

### Limitations of the review

This review has several limitations that should be acknowledged. It included studies that differed in design, participant selection and inclusion criteria. The exclusion of non-English publications may have introduced language and publication bias while the inclusion of one unpublished study prevented full appraisal of study methods.

Heterogeneity in analytic and reporting practices also restricted the conclusions we could draw. In OCT studies, segmentation methods and eye-specific analysis varied with some reports presenting data from only one eye. Oculomotor studies showed inconsistency in task design and outcome metrics limiting direct comparisons. Finally, although many OCT and oculomotor studies included visual acuity and color vision testing, these were rarely reported in detail.

## Conclusion

Current evidence indicates that quantitative oculomotor parameters have potential as non-invasive markers of neurodegeneration in HD. However, their suitability for clinical application remains uncertain due to methodological heterogeneity, small sample sizes, and limited longitudinal evaluation. Evidence for other ocular biomarkers, including OCT-derived retinal measures, is even less consistent; although many studies report abnormalities in HD, findings vary substantially across cohorts and protocols. Future work should prioritize standardized methodologies and long-term follow-up to determine the sensitivity of ocular biomarkers to disease progression and their responsiveness to therapeutic interventions.

## Data Availability

Not applicable.
